# Heterogeneity in family resilience among Chinese stroke patient-caregiver dyads: a latent profile analysis study

**DOI:** 10.3389/fpsyg.2026.1749638

**Published:** 2026-02-23

**Authors:** Jingjing Ma, Weifei Yu, Qihang Xu, Lu Shi, Yiqing Zhang

**Affiliations:** 1 Department of Nursing, Ningbo Medical Center Lihuili Hospital, Ningbo, China; 2 Department of Pharmacy, Ningbo Medical Center Lihuili Hospital, Ningbo, China; 3 Department of Rehabilitation Medicine, Ningbo Medical Center Lihuili Hospital, Ningbo, China

**Keywords:** caregivers, family, family resilience, latent profile analysis, stroke

## Abstract

**Background:**

While family resilience is a recognized determinant of adaptation following stroke, the distinct, empirically derived profiles of family resilience among Chinese stroke survivor-caregiver dyads have not been clearly delineated. Identifying these profiles and their determinants is crucial for developing targeted interventions.

**Objective:**

To identify latent profiles of family resilience and examine the socio-demographic and clinical factors associated with profile membership among stroke patient-caregiver dyads in China.

**Methods:**

In this cross-sectional study, a convenience sample of 773 stroke survivor-caregiver dyads was recruited from three hospitals in Zhejiang Province, China. Latent profile analysis (LPA) was conducted on the 20-item Family Resilience Questionnaire (FRQ). Multinomial logistic regression was used to determine factors associated with profile membership.

**Results:**

LPA supported a four-profile solution: Profile 1 “Low-Functioning Families” (22%), Profile 2 “Moderately Resilient - Low Cohesive Families” (24%), Profile 3 “Highly Resilient - Well-Functioning Families” (31%), and Profile 4 “High-Functioning - Optimistically Resilient Families” (24%). Multinomial logistic regression revealed that lower caregiver competence (higher FCTI scores) was strongly associated with profile membership (standardized aORs ranged from 2.58 to 43.19), whereas higher perceived social support (PSSS) was a significant protective factor (standardized aORs ranged from 0.03 to 0.19). Caregiver relationship and payment source were also significantly associated with profile membership.

**Conclusion:**

Family resilience among Chinese stroke families manifests in four distinct profiles, which are differentiated predominantly by caregiver competence and perceived social support. Our findings advocate for a precision family support paradigm, shifting from one-size-fits-all approaches to interventions tailored to distinct resilience profiles. Given the strong association, intervention programs should prioritize enhancing core caregiver competencies as a primary leverage point for building family resilience.

## Introduction

Stroke remains a major global public health challenge due to its high mortality and frequent long-term disability (Li Y. et al., 2024). Globally, the condition affects over 101 million people, with about 12.2 million new cases annually. China bears a particularly acute burden, accounting for nearly one-third of global cases and reporting 3.94 million new patients each year (GBD 2019 Stroke Collaborators, 2021; Wang et al., 2022).

The consequences of a stroke extend well beyond the individual, profoundly disrupting the entire family system. Informal caregivers, who are usually direct family, often endure considerable physical and emotional strain. This strain can, in turn, undermine both their own well-being and the quality of care provided (Lu et al., 2019). Within this challenging context, the concept of family resilience, which is defined as the family’s capacity to withstand and adapt to adversity by leveraging available resources, becomes paramount (Walsh, 2003). This adaptive capability is critical for maintaining household stability and positively influencing the survivor’s rehabilitation trajectory (Qureshi et al., 2023).

Despite broad recognition of its importance, family resilience is frequently treated in the literature as a uniform trait. Prevailing variable-centered approaches, by focusing on average scores, risk masking qualitatively different patterns of adaptation (Heerman et al., 2022; McKinley and Theall, 2021). A more pertinent question is whether families navigate post-stroke recovery in similar ways, or instead form distinct subgroups, each characterized by a unique configuration of strengths and vulnerabilities.

Latent Profile Analysis (LPA) provides a person-centered method to address this gap, shifting the focus from how much resilience a family has to what kind of resilience profile it exhibits. LPA has consistently identified heterogeneous subgroups, such as those with low, moderate, or high resilience, in contexts ranging from families of children with chronic illness (Dong et al., 2021) to caregivers of disabled older adults (Niu et al., 2025). Whether such distinct profiles exist among families facing stroke, however, remains unclear.

This study integrates two complementary theoretical frameworks. Walsh’s Family Resilience Framework outlines the core domains we profile: family belief systems, organizational patterns (directly reflected in caregiver competence), and communication processes (Walsh, 2003). Complementing this, the Social-Ecological Model (SEM) structures our investigation of predictors, guiding analysis across micro- (e.g., patient disability), meso- (e.g., social support), and macro-system (e.g., payment source) levels (Holt-Lunstad, 2018).

We thus pursue two primary objectives. First, using LPA on multidimensional family resilience data, we aim to identify distinct adaptation profiles within a sample of Chinese stroke survivor-caregiver dyads. Second, we examine how key demographic, clinical, and psychosocial factors are associated with membership in these profiles. Identifying such profiles lays a foundation for developing tailored interventions. Ultimately, profile-specific strategies could strengthen family resilience and improve long-term stroke care outcomes, particularly in high-burden settings like China.

## Methods

### Design

This study utilised a cross-sectional design. The reporting of this study follows the list of reports of observational studies (STROBE).

### Participants

Recruitment took place from September 1, 2023, to July 31, 2025, across three hospitals in Zhejiang Province, China. Our sampling strategy aimed to capture both acute and rehabilitative care phases by including two public tertiary hospitals—one in an eastern coastal city and another in a western inland city—alongside one private rehabilitation hospital. We used convenience sampling to recruit dyads of stroke survivors and their primary family caregivers. The latter were defined as unpaid family members (e.g., spouses, adult children, parents, or other relatives) identified by the survivor as the principal source of daily support during hospitalization (Denham et al., 2018). A total of 773 eligible inpatient dyads were ultimately enrolled.

Inclusion criteria for stroke survivors were: (1) diagnosis of stroke confirmed according to the Chinese Guidelines for the Main Subtypes of Cerebrovascular Diseases (2019); (2) age 18 years or older; (3) clinical stability, defined as no transfer to the intensive care unit, requirement for emergency interventions (e.g., intubation, vasopressor medication), or neurological deterioration within 72 h prior to enrollment; and (4) provision of voluntary informed consent.

Exclusion criteria for stroke survivors included: (1) presence of severe systemic comorbidities (e.g., metastatic cancer) or altered consciousness; and (2) withdrawal or refusal during the study process.

Inclusion criteria for primary family caregivers were: (1) identified as an unpaid family member (spouse, adult child, parent, or other relative) by the stroke survivor as the main source of daily support during hospitalization; (2) being actively involved in assisting with or supervising activities of daily living (e.g., feeding, positioning); (3) age 18 years or older; and (4) provision of voluntary informed consent.

Exclusion criteria for caregivers were: (1) hired professional caregivers or individuals providing only occasional, non-primary support; and (2) presence of a serious physical or mental health condition that could impede their caregiving capacity.

### Sample size

To ensure robust latent profile models, we conducted an *a priori* sample size estimation. Methodological guidelines for latent variable models with multiple continuous indicators recommend a sample exceeding 500 for reliable parameter estimation and profile identification (Sinha et al., 2021). Given the 20 observed indicators in our model, the final sample of 773 dyads satisfies this recommendation and exceeds the common 10:1 participant-to-variable heuristic, supporting the analytic adequacy of our sample.

### Measures

Trained assessors administered structured questionnaires during the survivors’ hospitalization. Demographic and clinical characteristics for both patients and caregivers were collected via a self-developed instrument capturing gender, age, education, time since diagnosis, employment status, primary medical payment method, and household income.

### Activities of daily living (ADL)

Functional independence among survivors was assessed using the Activities of Daily Living scale, which evaluates essential tasks like personal hygiene, dressing, feeding, and mobility. Scores range from 0 to 100, with higher values denoting greater independence. Following established cut-offs, scores of 61–99 indicate mild dependence (requiring occasional help), 41–60 moderate dependence (requiring substantial assistance), and ≤40 severe dependence (requiring full assistance for most activities) (Mlinac and Feng, 2016). Assessments were completed by a nurse during the hospital stay.

### Family resilience questionnaire (FRQ)

We measured family resilience using the 20-item FRQ developed by Bu and Liu (2019). This Chinese-specific scale comprises four dimensions: Perseverance (6 items), Harmony (5 items), Openness (5 items), and Supportiveness (4 items). All items use a 5-point Likert scale (1 = strongly disagree to 5 = strongly agree), yielding a total score between 20 and 100; higher scores reflect greater resilience. The scale has demonstrated strong psychometric properties, with a reported Cronbach’s *α* of 0.91 for the full scale (Bu and Liu, 2019). In the present sample, Cronbach’s *α* was 0.96.

### Family caregiver task inventory (FCTI)

Caregiver competence was evaluated with the 25-item FCTI, which assesses domains such as adapting to the caregiver role, responding to patient needs, managing personal emotions, assessing resources, and making life adjustments. Items are rated on a 5-point Likert scale. Notably, higher FCTI scores signify lower caregiver competence, reflecting greater difficulties in performing caregiving tasks. The Chinese version has shown high reliability (Cronbach’s *α* = 0.93) (Lee and Mok, 2011), which was consistent in our sample (*α* = 0.93).

### Perceived social support scale (PSSS)

The PSSS measured participants’ perceptions of support from family, friends, and significant others (Zimet et al., 1990). Its 12 items are rated on a 7-point Likert scale (1 = very strongly disagree to 7 = very strongly agree). Previous studies have established its high reliability (e.g., Cronbach’s *α* = 0.914) (Liu et al., 2016), a finding replicated here (*α* = 0.91).

### Outcome variable definition

The primary outcome was family resilience profile membership, a latent categorical variable generated from our analysis. Latent profile analysis (LPA) applied to the 20-item Family Resilience Questionnaire (FRQ) identified these subgroups. Selecting the optimal profile solution involved evaluating statistical fit indices alongside the theoretical coherence and distinctiveness of each class. Following model selection, each dyad was assigned to the profile for which it had the highest posterior probability, capturing its unique configuration of resilience attributes. These empirically derived profiles then functioned as the categorical outcome for subsequent predictive modeling.

### Statistical analysis

Analyses proceeded in two sequential stages: latent profile identification followed by predictor examination.

The first stage used Latent Profile Analysis (LPA) in Mplus 8.3 to identify unobserved subgroups based on responses to the 20 FRQ items. We estimated models specifying one through six profiles. Model selection involved evaluating statistical fit indices, with lower values on the AIC, BIC, and aBIC preferred, and higher entropy values indicating more distinct profiles. We also assessed classification precision using entropy, with values ≥ 0.80 deemed acceptable, and used the Lo–Mendell–Rubin adjusted likelihood ratio test (LMRT) and the bootstrap likelihood ratio test (BLRT) to statistically compare a k-profile model against a k-1 profile model. When statistical indices were inconclusive, final model choice emphasized theoretical interpretability and clinical relevance, favoring the more parsimonious solution.

The second stage, performed in SPSS 26.0, involved descriptive and inferential analyses. Descriptive statistics characterized the sample, with continuous variables reported as mean ± standard deviation or median (interquartile range) based on distribution, and categorical variables as frequencies (percentages). Bivariate associations between profile membership and candidate predictors were tested using chi-square tests for categorical variables. To identify factors independently associated with profile membership, we performed multinomial logistic regression. For this model, continuous predictors—specifically scores on the Family Caregiver Task Inventory (FCTI) and the Perceived Social Support Scale (PSSS)—were converted to *Z*-scores to facilitate comparison of effect magnitudes. Statistical significance was set at *p* < 0.05 (two-tailed) for all tests.

## Results

### Sample characteristics

A total of 773 patient-caregiver dyads completed the study. The patient cohort was predominantly male (57.70%), whereas caregivers were mostly female (61.06%). Full demographic and clinical characteristics for both groups are detailed in Table 1.

**Table 1:** Demographic and clinical characteristics of stroke survivors by resilience profile.

Characteristic	Total (*n* = 773)	1 (*n* = 168)	2 (*n* = 186)	3 (*n* = 237)	4 (*n* = 182)	Statistic	*p*
Gender, *n* (%)						*χ*^2^ = 23.29	<0.001
Male	446 (57.70)	82 (48.81)	101 (54.30)	131 (55.27)	132 (72.53)		
Female	327 (42.30)	86 (51.19)	85 (45.70)	106 (44.73)	50 (27.47)		
Age, *n* (%)						*χ*^2^ = 22.05	0.009
18–40 years	78 (10.09)	18 (10.71)	22 (11.83)	26 (10.97)	12 (6.59)		
40–60 years	244 (31.57)	62 (36.90)	60 (32.26)	71 (29.96)	51 (28.02)		
60–80 years	278 (35.96)	55 (32.74)	79 (42.47)	81 (34.18)	63 (34.62)		
> 80 years	173 (22.38)	33 (19.64)	25 (13.44)	59 (24.89)	56 (30.77)		
Educational, *n* (%)						*χ*^2^ = 81.92	<0.001
Primary or below	473 (61.19)	117 (69.64)	141 (75.81)	139 (58.65)	76 (41.76)		
Middle school	183 (23.67)	43 (25.60)	29 (15.59)	57 (24.05)	54 (29.67)		
High school/College	75 (9.70)	7 (4.17)	16 (8.60)	26 (10.97)	26 (14.29)		
Bachelor’s or above	42 (5.43)	1 (0.60)	0 (0.00)	15 (6.33)	26 (14.29)		
Occupational, *n* (%)						*χ*^2^ = 97.06	<0.001
Employed	196 (25.36)	42 (25.00)	55 (29.57)	49 (20.68)	50 (27.47)		
Retirement	212 (27.43)	26 (15.48)	20 (10.75)	77 (32.49)	89 (48.90)		
Unemployed/Other	365 (47.22)	100 (59.52)	111 (59.68)	111 (46.84)	43 (23.63)		
Cost, *n* (%)						*χ*^2^ = 105.45	<0.001
Rural medical insurance	340 (43.98)	39 (23.21)	66 (35.48)	111 (46.84)	124 (68.13)		
Urban medical insurance	274 (35.45)	61 (36.31)	86 (46.24)	82 (34.60)	45 (24.73)		
Out-of-pocket/Commercial	159 (20.57)	68 (40.48)	34 (18.28)	44 (18.57)	13 (7.14)		
Income, *n* (%)						*χ*^2^ = 142.04	<0.001
< 3,000 CNY	421 (54.46)	118 (70.24)	143 (76.88)	112 (47.26)	48 (26.37)		
3,000–8,000 CNY	302 (39.07)	50 (29.76)	42 (22.58)	108 (45.57)	102 (56.04)		
≥ 8,000 CNY	50 (6.47)	0 (0.00)	1 (0.54)	17 (7.17)	32 (17.58)		
ADL, *n* (%)						*χ*^2^ = 84.22	<0.001
Independent/Mild	324 (41.91)	91 (54.17)	101 (54.30)	74 (31.22)	58 (31.87)		
Moderate	227 (29.37)	59 (35.12)	57 (30.65)	66 (27.85)	45 (24.73)		
Severe	222 (28.72)	18 (10.71)	28 (15.05)	97 (40.93)	79 (43.41)		

Z, Mann–Whitney test; *χ*^2^, Chi-square test; M, Median; Q₁, 1st Quartile; Q₃, 3st Quartile.

### Identifying family resilience profiles

Analysis of responses to the Family Resilience Questionnaire using latent profile analysis (LPA) identified distinct subgroups. Table 2 presents model fit indices for one- through five-profile solutions, with descriptive statistics for all FRQ items available in Table 3.

**Table 2:** Demographic characteristics of primary stroke caregivers (*N* = 773).

Characteristic	Total (*n* = 773)	1 (*n* = 168)	2 (*n* = 186)	3 (*n* = 237)	4 (*n* = 182)	Statistic	*p*
Gender, *n* (%)						*χ*^2^ = 22.58	<0.001
Male	301 (38.94)	83 (49.40)	75 (40.32)	97 (40.93)	46 (25.27)		
Female	472 (61.06)	85 (50.60)	111 (59.68)	140 (59.07)	136 (74.73)		
Age, *n* (%)						*χ*^2^ = 67.55	<0.001
18–40 years	268 (34.67)	86 (51.19)	58 (31.18)	89 (37.55)	35 (19.23)		
40–60 years	272 (35.19)	28 (16.67)	80 (43.01)	70 (29.54)	94 (51.65)		
60–80 years	194 (25.10)	44 (26.19)	44 (23.66)	63 (26.58)	43 (23.63)		
Over 80 years	39 (5.05)	10 (5.95)	4 (2.15)	15 (6.33)	10 (5.49)		
Educational, *n* (%)						*χ*^2^ = 29.42	<0.001
Primary or below	134 (17.34)	17 (10.12)	27 (14.52)	52 (21.94)	38 (20.88)		
Middle school	218 (28.20)	46 (27.38)	58 (31.18)	56 (23.63)	58 (31.87)		
High school/College	155 (20.05)	25 (14.88)	40 (21.51)	50 (21.10)	40 (21.98)		
Bachelor’s or above	266 (34.41)	80 (47.62)	61 (32.80)	79 (33.33)	46 (25.27)		
Relationship, *n* (%)						*χ*^2^ = 27.07	<0.001
Spouse	295 (38.16)	50 (29.76)	58 (31.18)	91 (38.40)	96 (52.75)		
Direct family	394 (50.97)	97 (57.74)	110 (59.14)	117 (49.37)	70 (38.46)		
Other	84 (10.87)	21 (12.50)	18 (9.68)	29 (12.24)	16 (8.79)		
Work, *n* (%)						*χ*^2^ = 30.23	<0.001
Employed	391 (50.58)	98 (58.33)	94 (50.54)	117 (49.37)	82 (45.05)		
Retired	164 (21.22)	15 (8.93)	34 (18.28)	57 (24.05)	58 (31.87)		
Others	218 (28.20)	55 (32.74)	58 (31.18)	63 (26.58)	42 (23.08)		
Time, *n* (%)						*χ*^2^ = 145.01	<0.001
≤3 weeks	386 (49.94)	125 (74.40)	88 (47.31)	110 (46.41)	63 (34.62)		
4–5 weeks	194 (25.10)	21 (12.50)	13 (6.99)	77 (32.49)	83 (45.60)		
≥ 6 weeks	193 (24.97)	22 (13.10)	85 (45.70)	50 (21.10)	36 (19.78)		
FCTI, M (Q₁, Q₃)	1.76 (1.16, 3.04)	3.22 (3.00, 3.40)	1.76 (1.16, 2.84)	1.60 (1.24, 2.20)	1.12 (0.89, 1.48)	*χ*^2^ = 342.83^#^	<0.001
PSSS, M (Q₁, Q₃)	3.36 (2.28, 5.25)	3.24 (3.04, 3.40)	1.80 (1.16, 2.97)	3.44 (1.88, 5.17)	6.00 (5.10, 6.58)	*χ*^2^ = 401.97^#^	<0.001

ADL, Activities of Daily Living; CNY, Chinese Yuan; FCTI, Family Caregiver Task Inventory; PSSS, Perceived Social Support Scale; Q₁, First Quartile; Q₃, Third Quartile. FCTI and PSSS scores are presented on their original measurement scales (5-point and 7-point Likert scales, respectively) and should not be directly compared in magnitude. ⁰Kruskal-Wallis H test statistic.

**Table 3:** Descriptive statistics of FRQ.

Items	Variable	Average item score	Minimum Value	Maximum Value	Median value
1	Our family members are motivated and strive to improve.	3.74 ± 0.86	1	5	4
2	At home, we can speak our minds openly without holding back.	3.78 ± 0.84	1	5	4
3	Most people in our family enjoy learning new things.	3.35 ± 1.26	1	5	4
4	Friends often come to my home to visit.	3.38 ± 1.29	1	5	4
5	People in our family are optimists.	3.32 ± 1.25	1	5	3
6	Even anger and outbursts do not affect our family ties.	3.36 ± 1.13	1	5	4
7	Our family believes we can grow through adversity.	3.37 ± 1.24	1	5	3
8	We are honest with each other.	2.74 ± 1.67	1	5	3
9	When our family encounters difficulties, relatives and friends always take the initiative to help us.	3.73 ± 0.74	1	5	4
10	Family members show love for each other in their own ways.	2.81 ± 1.46	1	5	3
11	When I face difficulties, my family helps by offering ideas and solutions.	3.36 ± 1.27	1	5	4
12	Everyone in our family plays an important role.	3.36 ± 1.26	1	5	4
13	When facing problems, our family always finds a way to solve them.	3.39 ± 1.25	1	5	4
14	I can talk to my family when I’m hurt by something outside.	3.70 ± 0.83	1	5	4
15	People in our family are not easily overwhelmed by difficulties.	3.28 ± 1.26	1	5	3
16	When facing difficulties, our family members discuss how to cope together.	2.92 ± 1.41	1	5	3
17	Our family enjoys spending pleasant time sitting together.	2.75 ± 1.66	1	5	3
18	When difficulties arise, our family believes we can overcome them ourselves.	3.35 ± 1.25	1	5	3
19	Our family becomes more resilient when facing setbacks.	3.19 ± 1.22	1	5	3
20	When making important decisions, family members seek each other’s opinions.	2.84 ± 1.36	1	5	3

A four-profile solution was ultimately selected. Although information criteria (AIC, BIC, aBIC) continued to decrease up to five profiles, the non-significant Lo–Mendell–Rubin and Bootstrap Likelihood Ratio Tests (*p* = 0.8046) for the five-profile model indicated that the added complexity was not statistically justified. The four-profile model provided excellent classification certainty (entropy = 0.984) and represented a significant improvement over models with fewer classes (*p* < 0.001), while yielding subgroups that were both theoretically interpretable and clinically meaningful (see Table 4).

**Table 4:** Model fit indices for latent profile analysis of family resilience in stroke survivors (*N* = 773).

Number of Profiles	AIC	BIC	aBIC	Entropy	LMRT (*p*-value)	BLRT (*p*-value)	Profile Probabilities (n)
1-Class	49565.877	49751.888	49624.87	—	—	—	1
2-Class	39637.803	39921.47	39727.766	0.999	<0.001	<0.001	0.47/0.53
3-Class	36328.100	36709.423	36449.035	0.985	<0.001	<0.001	0.46/0.30/0.24
**4-Class**	**32984.179**	**33463.158**	**33136.085**	**0.984**	**<0.001**	**<0.001**	**0.22/0.24/0.31/0.24**
5-Class	31986.018	32562.653	32168.895	0.979	<0.8046	<0.8045	0.22/0.24/0.19/0.20/0.15

The optimal 4-class solution is highlighted in bold. AIC, Akaike Information Criterion; BIC, Bayesian Information Criterion; aBIC, Sample-Size Adjusted BIC; LMRT, Lo–Mendell–Rubin Adjusted Likelihood Ratio Test; BLRT, Bootstrap Likelihood Ratio Test. Lower values on AIC, BIC, and aBIC indicate better fit. Higher entropy values (closer to 1) indicate clearer classification. Significant p-values for LMRT and BLRT suggest that the k-class model fits significantly better than the (k-1)-class model.

### Characterization of resilience profiles

**Figure 1:**
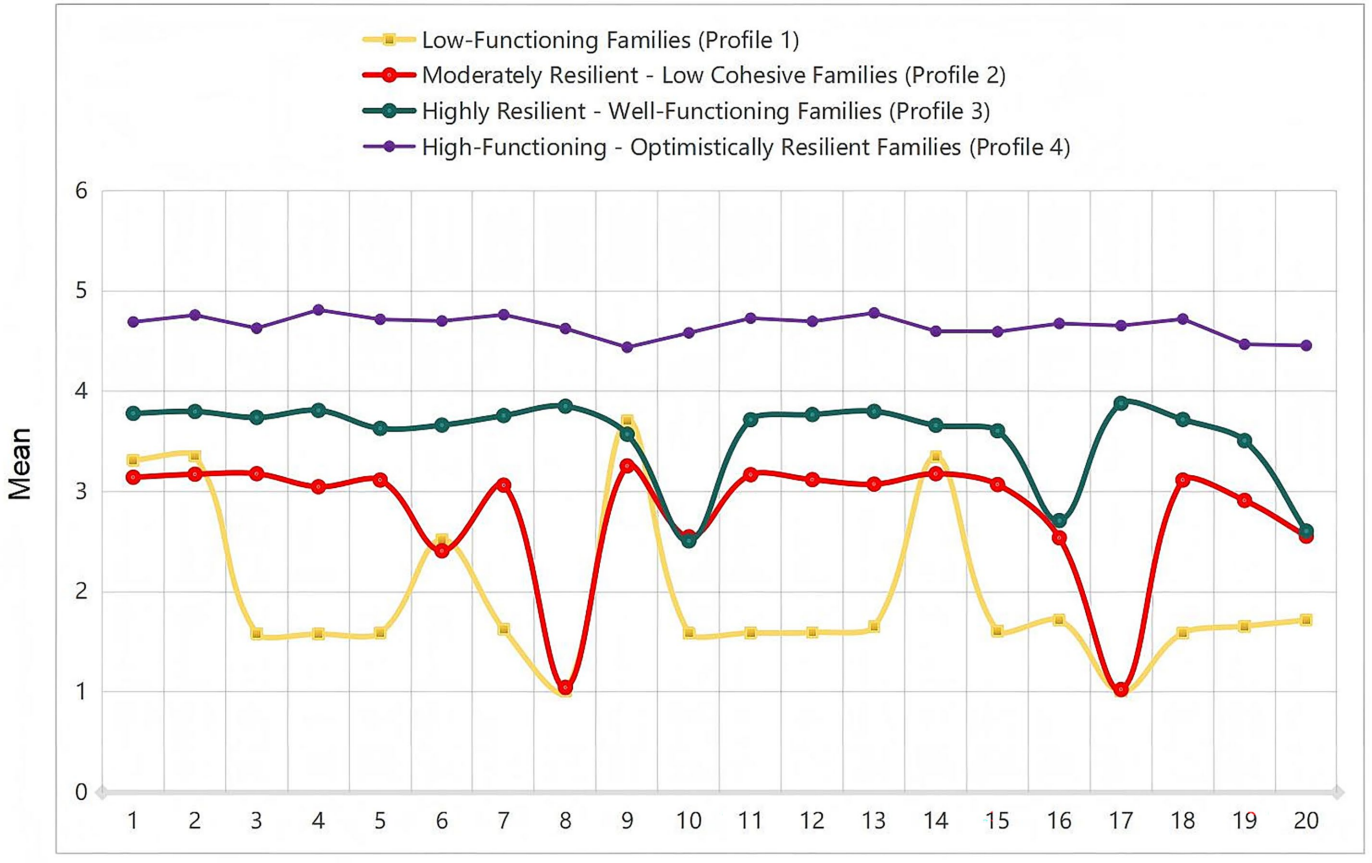
Latent profiles of family resilience based on the 20-item Family Resilience Questionnaire (FRQ) scores among stroke patient-caregiver dyads (*N* = 773).

These four profiles corresponded to distinct patterns of family adaptation (Figure 1):

Profile 1: Low-Functioning Families (22%) demonstrated consistently low scores across all resilience domains, indicating pervasive difficulties in family adaptation.
Profile 2: Moderately Resilient – Low Cohesive Families (24%) presented a mixed pattern, characterized by relatively preserved pragmatic functioning alongside notable weaknesses in family harmony and shared belief systems.
Profile 3: Highly Resilient – Well-Functioning Families (31%) constituted the largest subgroup and exhibited strong, balanced resilience capacities across most domains.
Profile 4: High-Functioning – Optimistically Resilient Families (24%) were distinguished by exceptionally high scores, particularly on items reflecting optimism, a growth mindset, and the active utilization of external resources.

### Predictors of profile membership

Initial bivariate tests revealed significant associations between profile membership and every demographic and clinical variable examined (*p* < 0.01; see Table 1). As shown in Table 5, key study variables were interrelated: family resilience scores correlated negatively with caregiver difficulty (*r* = −0.686, *p* < 0.01) and positively with perceived social support (*r* = 0.638, *p* < 0.01), while caregiver difficulty and social support were themselves inversely correlated (*r* = −0.265, *p* < 0.01).

**Table 5:** Correlations (r) between perceived social support, family resilience, and caregiver capacity.

Variable	FRQ	FCTI	PSSS
FRQ	1		
FCTI	−0.686**	1	
PSSS	0.638**	−0.265**	1

The Family Resilience Scale (FRQ) assesses family resilience. The Family Caregiver Task Inventory (FCTI) is used to evaluate the caregiver’s abilities. The Perceived Social Support Scale (PSSS) is a scale used to assess an individual’s perceived level of social support. **p-value < 0.01.

Results from the multinomial logistic regression, which used Profile 1 as the reference category, identified several independent predictors of profile membership (Table 6). Caregiver competence (operationalized by FCTI scores) and perceived social support (PSSS) were among the strongest predictors. Patient gender, primary payment source, caregiver relationship to the patient, daily hours of care, and patient functional status also contributed significantly to the model (all *p* < 0.05). This multivariate analysis suggests that these factors offer unique explanatory power in differentiating between family resilience profiles, beyond their initial bivariate associations.

**Table 6:** Multivariable logistic regression analysis of factors associated with family resilience profiles (*N* = 773).

Variable	Profile 2 vs. Profile 1 (Ref.)			Profile 3 vs. Profile 1 (Ref.)			Profile 4 vs. Profile 1 (Ref.)		
	aOR (95% CI)	Wald	*p*	aOR (95% CI)	Wald	*p*	aOR (95% CI)	Wald	*p*
FCTI	43.19 (19.41, 96.13)	85.1	< 0.001	8.55 (4.31, 16.93)	37.83	< 0.001	2.58 (1.62, 4.10)	15.98	< 0.001
PSSS	0.10 (0.04, 0.27)	20.1	< 0.001	0.03 (0.01, 0.07)	62.88	< 0.001	0.19 (0.13, 0.30)	55.01	< 0.001
Patient’s gender									
Male	0.55 (0.24, 1.29)	1.9	0.169	0.58 (0.26, 1.28)	1.83	0.177	0.48 (0.26, 0.91)	5.03	0.025
Female*	–	–	–	–	–	–	–	–	–
Payment source									
Rural Resident Insurance	3.08 (0.78, 12.16)	2.57	0.109	5.85 (1.54, 22.22)	6.74	0.009	2.94 (0.94, 9.21)	3.42	0.064
Urban Employee Insurance	1.64 (0.43, 6.35)	0.52	0.472	4.74 (1.26, 17.87)	5.29	0.021	2.52 (0.80, 8.02)	2.47	0.116
Out-of-pocket/Commercial *	–	–	–	–	–	–	–	–	–
Caregiver’s relationship									
Spouse	0.12 (0.02, 0.55)	7.29	0.007	0.09 (0.02, 0.35)	11.71	0.001	0.20 (0.08, 0.54)	10.11	0.001
Direct Family	0.25 (0.05, 1.23)	2.89	0.089	0.27 (0.07, 1.09)	3.39	0.066	0.29 (0.11, 0.78)	6.07	0.014
Other*	–	–	–	–	–	–	–	–	–
Daily care time									
≤ 3 weeks	0.84 (0.27, 2.54)	0.1	0.75	0.36 (0.14, 0.93)	4.45	0.035	0.87 (0.41, 1.87)	0.13	0.722
4–5 weeks	0.69 (0.20, 2.39)	0.34	0.558	0.16 (0.05, 0.49)	10.27	0.001	0.87 (0.41, 1.85)	0.12	0.724
≥ 6 weeks*	–	–	–	–	–	–	–	–	–
Patient’s ADL									
Independent/Mild	2.33 (0.79, 6.90)	2.33	0.127	2.23 (0.85, 5.81)	2.68	0.101	0.76 (0.39, 1.51)	0.6	0.44
Moderate	3.15 (1.03, 9.65)	4.06	0.044	1.92 (0.71, 5.22)	1.63	0.202	1.04 (0.51, 2.12)	0.01	0.919
Severe*	–	–	–	–	–	–	–	–	–

*The reference category. aOR, Adjusted Odds Ratio; CI, Confidence Interval. The model was adjusted for all variables listed in the table, as well as other covariates (including patient and caregiver age, education, and occupational status) that were not statistically significant and are omitted for clarity. FCTI and PSSS were entered as standardized *Z*-scores; their aORs represent the change in odds associated with a one-standard-deviation increase and are directly comparable. Wald statistics are provided for significant and key variables.

## Discussion

### Heterogeneity in family resilience profiles

Latent profile analysis identified four distinct family resilience profiles among stroke patient-caregiver dyads. This result directly challenges the notion of adaptation as a uniform construct distributed along a simple high-low continuum, confirming instead the multidimensional and heterogeneous nature of family resilience (Dong et al., 2021; Herbers et al., 2020). The emergence of these qualitatively different profiles supports a person centered perspective, suggesting that families configure their unique strengths and vulnerabilities into specific patterns of adaptation.

### Characterization of resilience profiles

The four profiles reflect distinct configurations of resilience strengths and vulnerabilities, patterns which align closely with established theories of family adaptation.

Profile 1 (Low-Functioning Families) presents a complex clinical picture where preserved foundational capacities coexist with critical deficits in key processes. While these families maintain basic abilities in problem-solving (Item 1: 3.315) and role recognition (Item 2: 3.353), they struggle profoundly with emotional communication and conflict management (Item 8: 1.012). This pattern illustrates how core relational processes can falter even when some instrumental functions remain intact, consistent with Walsh’s emphasis on communication as the essential conduit for adaptive transformation under stress (Walsh, 2016).

Profile 2 (Moderately Resilient-Low Cohesive Families) reveals a pronounced dissociation between practical function and emotional connection. Competence in collaborative tasks (Item 1: 3.144; Item 4: 3.049) contrasts sharply with marked difficulties in expressing affection (Item 6: 2.41) and managing interpersonal dynamics (Item 8: 1.048). This fragmentation mirrors what Henry et al. describe as “functional fragmentation” (Henry et al., 2015), where families maintain operational competence while experiencing emotional disengagement, potentially compromising long-term adaptation.

The Chinese cultural context offers a lens through which to interpret this profile. Traditional values emphasizing harmony and filial obligation often drive pragmatic, task-focused caregiving during health crises (Yang et al., 2025). However, these same norms can suppress the open expression of distress to maintain surface harmony (Li Q. et al., 2024). In such settings, families may thus demonstrate functional caregiving competence while internalizing significant emotional strain. This culturally informed pattern of “high pragmatism, low emotional expressiveness” helps explain the coexistence of moderate resilience and low cohesion seen in Profile 2.

Profile 3 (Highly Resilient-Well-Functioning Families) demonstrates robust and integrated functioning across most domains. Relative to Profile 4, however, this group appears more inwardly focused, exhibiting comparatively lower scores on growth-through-adversity (Item 10: 2.509) and external social engagement (Item 20: 2.607). This configuration aligns with Black and Lobo’s observation that some well-functioning families prioritize strong internal cohesion while maintaining more bounded external connections (Black and Lobo, 2008).

Profile 4 (High-Functioning-Optimistically Resilient Families) distinguishes itself through exceptional performance across all dimensions, particularly excelling in proactive growth (Item 10: 4.584) and active social resource utilization (Item 20: 4.458). This profile embodies Walsh’s concept of transformative resilience, in which families not only withstand crisis but also experience growth and re-organization (Walsh, 2016). Their capacity to integrate pragmatic functioning with emotional attunement and optimistic outreach represents the most comprehensive realization of adaptive family processes identified in this sample.

### The central role of caregiver competence and social support

The analysis positions caregiver competence and perceived social support as central, distinguishing factors among the resilience profiles. Notably, membership in Profile 2 relative to Profile 1 was strongly associated with lower caregiver competence. This link resonates with the Family Stress Model (Pearlin et al., 1990), where difficulties in performing core caregiving tasks can deplete a family’s internal resources, fostering an adaptation pattern that sustains practical functioning at the expense of emotional cohesion.

Higher levels of perceived social support, conversely, were uniquely associated with membership in the higher-functioning profiles (Profiles 3 and 4). This finding aligns with the buffering hypothesis (Cohen and Wills, 1985), positing that robust external support networks provide essential resources that help maintain family integrity and facilitate the proactive, optimistic outlook observed in the most resilient profiles. Within our model, these two factors operated as related yet distinct components of the family’s resource ecosystem.

### Influence of caregiver and patient characteristics

Several demographic and clinical characteristics further delineated profile membership. Spousal caregivers showed a different pattern of association than direct family, being less prevalent in the most resilient Profile 4. This result underscores the distinct emotional dynamics of spousal caregiving (Pinquart and Sörensen, 2011), wherein the relationship’s intensity and the risk of role engulfment (Panourgia et al., 2022; Skaff and Pearlin, 1992) may shape a family’s adaptive response, particularly in developing the optimistic, growth-oriented resilience epitomized by Profile 4.

Specific patient characteristics also proved influential. For instance, families of patients with moderate functional dependence were more frequently classified in Profile 2 than in Profile 1, a pattern consistent with the competence-press model (Rizeq et al., 2021). This indicates that moderate care demands might be sufficient to mobilize a family’s problem-solving capacities while simultaneously generating interpersonal strains that erode emotional unity. Furthermore, families with male patients were less represented in Profile 4, possibly reflecting gendered patterns in illness response and support-seeking that merit further investigation (Wang et al., 2025).

### The influence of income

A strong bivariate association was observed between household monthly income and resilience profile membership (*χ*^2^ = 142.04, *p* < 0.001). Notably, households with a monthly income of ≥8,000 CNY were almost absent from the two lower-resilience profiles (Profile 1: 0%; Profile 2: 0.54%) but constituted a markedly higher proportion of Profile 4 (17.58%). This distribution pattern indicates a close link between higher financial resources and more resilient family configurations.

Drawing on theoretical frameworks, economic capital may function as a critical contextual resource that can facilitate adaptation (Schüz et al., 2016). Beyond alleviating immediate financial strain, it may help stabilize a caregiver’s employment status and preserve opportunities for social engagement, thereby fostering a family climate more conducive to effective communication, collaborative problem-solving, and shared positive beliefs (Conger and Donnellan, 2007). These potential pathways are consistent with the Family Stress Model, which posits economic pressure as a salient factor interacting with family processes.

Nevertheless, the near-total separation of higher-income households from the low-resilience profiles rendered this variable unstable for estimation in our multivariate model. Future research should employ more refined economic indicators and larger, socioeconomically diverse samples to clarify the unique contribution of financial resources to family resilience trajectories.

### Clinical implications and intervention strategies

This study applied latent profile analysis to reveal four distinct configurations of family resilience, demonstrating that structural heterogeneity characterizes how families adapt following a stroke. The identified profiles enable a shift toward precision family support, where interventions must be tailored to specific patterns of strengths and deficits. For “Low-Functioning Families” (Profile 1), interventions should initiate a crisis management and resource linkage protocol, prioritizing respite care and basic financial navigation to alleviate their overwhelming burden. For “Moderately Resilient but Low-Cohesive Families” (Profile 2), brief family counseling focused on emotional validation and safe expression is indicated, directly targeting their core deficits in specific emotional communication and conflict management items. For the highly resilient families (Profiles 3 and 4), the goal is to preserve robust functioning through preventive support. Specifically, for “High-Functioning-Optimistically Resilient Families” (Profile 4), their strengths can be leveraged by formally engaging them as peer support mentors within the care community. This stratified approach moves beyond one-size-fits-all support and is essential for effectively promoting sustainable adaptation and post-traumatic growth in stroke families (Liu et al., 2025; McCurley et al., 2019).

A recent longitudinal study investigating the trajectory of family resilience during the first 6 months post-stroke (Zhang et al., 2023) highlights the need to consider both temporal dynamics and structural heterogeneity for a comprehensive understanding of post-stroke family adaptation. Building on the profiles established in the present study, future longitudinal research should examine the stability of these resilience configurations over time.

## Conclusion

This study identified four distinct profiles of family resilience among stroke patient-caregiver dyads, revealing that adaptation is best understood not as a linear continuum but as heterogeneous configurations of strengths and vulnerabilities. Key factors, including caregiver competence, perceived social support, caregiver relationship dynamics, and patient functional status, proved instrumental in differentiating these profiles. The results argue for a paradigm shift in family-centered stroke care: from generic support to a precision intervention model where initial assessment of a family’s resilience profile guides subsequent action. For instance, families in the low-functioning profile require immediate resource linkage and practical stabilization, whereas those in the moderately resilient but low-cohesive profile may benefit most from brief counseling targeting emotional communication. By aligning support strategies with these empirically derived patterns, clinical practice offers the potential not only alleviate burden but also actively foster post-stroke growth and sustained adaptation.

## Limitations

This study has several limitations that should be considered. First, the cross-sectional design precludes the establishment of causal relationships between identified factors and family resilience profiles. Second, although participants were recruited from multiple hospitals, all were located within a single province in China, which may restrict the generalizability of the findings. Third, the reliance on self-reported measures introduces the possibility of common method bias. Furthermore, the uneven distribution of certain characteristics, particularly the absence of high-income (≥8,000 CNY) families in the lowest-resilience profile, likely affected the stability of multivariate estimates. Finally, several potentially relevant variables were not assessed, such as stroke severity measured by a standardized scale (e.g., NIHSS), objective indicators of financial burden, and availability of community-based resources. Future longitudinal studies spanning multiple regions, with more balanced socioeconomic representation and the inclusion of objective clinical and contextual measures, are needed to validate the identified profiles and examine their temporal dynamics.

## Data Availability

The data supporting this study are available from the corresponding author upon reasonable request.

## References

[ref1] Black K. Lobo M. (2008). A conceptual review of family resilience factors. J. Fam. Nurs. 14, 33–55. doi: 10.1177/1074840707312237 18281642

[ref2] Bu T. Liu H. (2019). Development of the family resilience questionnaire. Psychol. Tech. Appl. 7, 173–182. doi: 10.16842/j.cnki.issn2095-5588.2019.03.006

[ref3] Cohen S. Wills T. A. (1985). Stress, social support, and the buffering hypothesis. Psychol. Bull. 98, 310–357. doi: 10.1037/0033-2909.98.2.310 3901065

[ref4] Conger R. D. Donnellan M. B. (2007). An interactionist perspective on the socioeconomic context of human development. Annu. Rev. Psychol. 58, 175–199. doi: 10.1146/annurev.psych.58.110405.085551, 16903807

[ref5] Denham A. M. J. Baker A. L. Spratt N. Guillaumier A. Wynne O. Turner A. . (2018). The unmet needs of informal carers of stroke survivors: a protocol for a systematic review of quantitative and qualitative studies. BMJ Open 8:e019571. doi: 10.1136/bmjopen-2017-019571, 29391371 PMC5878248

[ref6] Dong C. Wu Q. Pan Y. Yan Q. Xu R. Zhang R. (2021). Family resilience and its association with psychosocial adjustment of children with chronic illness: a latent profile analysis. J. Pediatr. Nurs. 60, e6–e12. doi: 10.1016/j.pedn.2021.02.010, 33622641

[ref7] GBD 2019 Stroke Collaborators (2021). Global, regional, and national burden of stroke and its risk factors, 1990-2019: a systematic analysis for the global burden of disease study 2019. Lancet Neurol 20, 795–820. doi: 10.1016/S1474-4422(21)00252-0, 34487721 PMC8443449

[ref8] Heerman W. J. Samuels L. R. González Peña T. van Wyk C. Mayberry L. S. Lounds Taylor J. . (2022). Family resilience and childhood obesity among children exposed to adverse childhood experiences in a national survey. Obes. Sci. Pract. 8, 3–11. doi: 10.1002/osp4.497, 35127118 PMC8804940

[ref9] Henry C. S. Morris A. S. Harrist A. W. (2015). Family resilience: moving into the third wave. Fam. Relat. 64, 22–43. doi: 10.1111/fare.12106

[ref10] Herbers J. E. Cutuli J. J. Keane J. N. Leonard J. A. (2020). Childhood homelessness, resilience, and adolescent mental health: a prospective, person-centered approach. Psychol. Sch. 57, 1830–1844. doi: 10.1002/pits.22331, 33424042 PMC7792983

[ref11] Holt-Lunstad J. (2018). Why social relationships are important for physical health: a systems approach to understanding and modifying risk and protection. Annu. Rev. Psychol. 69, 437–458. doi: 10.1146/annurev-psych-122216-011902 29035688

[ref12] Lee R. L. T. Mok E. S. B. (2011). Evaluation of the psychometric properties of a modified Chinese version of the caregiver task inventory--refinement and psychometric testing of the Chinese caregiver task inventory: a confirmatory factor analysis. J. Clin. Nurs. 20, 3452–3462. doi: 10.1111/j.1365-2702.2011.03729.x 21707805

[ref13] Li Y. Li N. Zhou Y. Li L. (2024). Predicting ineffective thrombolysis in acute ischemic stroke with clinical and biochemical markers. Sci. Rep. 14:13424. doi: 10.1038/s41598-024-64413-w, 38862629 PMC11166982

[ref14] Li Q. Luo C. Ye J. Bian Z. Sun W. Zhou M. . (2024). Relationship between illness uncertainty and family resilience among caregivers of stroke patients in Chinese nuclear families: the mediating role of perceived stress. Patient Prefer. Adherence 18, 1095–1105. doi: 10.2147/PPA.S463562, 38854479 PMC11162204

[ref15] Liu L. Gou Z. Zuo J. (2016). Social support mediates loneliness and depression in elderly people. J. Health Psychol. 21, 750–758. doi: 10.1177/1359105314536941, 24925547

[ref16] Liu S. Zhou W. Wu X. Ding J. Liu Y. Shao H. . (2025). Post-traumatic growth in young and middle-aged patients with stroke: a qualitative systematic review and meta-synthesis. BMC Psychol. 13:967. doi: 10.1186/s40359-025-03321-8, 40859311 PMC12382294

[ref17] Lu Q. Mårtensson J. Zhao Y. Johansson L. (2019). Living on the edge: family caregivers’ experiences of caring for post-stroke family members in China: a qualitative study. Int. J. Nurs. Stud. 94, 1–8. doi: 10.1016/j.ijnurstu.2019.02.016 30928717

[ref18] McCurley J. L. Funes C. J. Zale E. L. Lin A. Jacobo M. Jacobs J. M. . (2019). Preventing chronic emotional distress in stroke survivors and their informal caregivers. Neurocrit. Care. 30, 581–589. doi: 10.1007/s12028-018-0641-6, 30421266 PMC6958510

[ref19] McKinley C. E. Theall K. P. (2021). Weaving healthy families program: promoting resilience while reducing violence and substance use. Res. Soc. Work. Pract. 31, 476–492. doi: 10.1177/1049731521998441, 34257501 PMC8274525

[ref20] Mlinac M. E. Feng M. C. (2016). Assessment of activities of daily living, self-care, and independence. Arch. Clin. Neuropsychol. 31, 506–516. doi: 10.1093/arclin/acw049, 27475282

[ref21] Niu Y. Xu Z. Huang J. Guo S. Lou T. Bai X. . (2025). A latent class analysis of resilience and its relationship with care burden and psychological distress in family caregivers of older adults with disability. Nurs. Health Sci. 27:e70069. doi: 10.1111/nhs.70069, 40064541

[ref22] Panourgia C. Wezyk A. Ventouris A. Comoretto A. Taylor Z. Yankouskaya A. (2022). Individual factors in the relationship between stress and resilience in mental health psychology practitioners during the COVID-19 pandemic. J. Health Psychol. 27, 2613–2631. doi: 10.1177/13591053211059393, 34875921 PMC9483698

[ref23] Pearlin L. I. Mullan J. T. Semple S. J. Skaff M. M. (1990). Caregiving and the stress process: an overview of concepts and their measures. Gerontologist 30, 583–594. doi: 10.1093/geront/30.5.583, 2276631

[ref24] Pinquart M. Sörensen S. (2011). Spouses, adult children, and children-in-law as caregivers of older adults: a meta-analytic comparison. Psychol. Aging 26, 1–14. doi: 10.1037/a0021863, 21417538 PMC4449135

[ref25] Qureshi A. Swain N. Aldabe D. Hale L. (2023). Exploring challenges affecting resilience in carers of stroke survivors: a qualitative descriptive study. Disabil. Rehabil. 45, 3696–3704. doi: 10.1080/09638288.2022.2135774, 36269117

[ref26] Rizeq J. Korczak D. J. Cost K. T. Anagnostou E. Charach A. Monga S. . (2021). Vulnerability pathways to mental health outcomes in children and parents during COVID-19—PubMed. Curr. Psychol. 19, 1–11. doi: 10.1007/s12144-021-02459-z PMC8603653 34815638

[ref27] Schüz B. Westland J. N. Wurm S. Tesch-Römer C. Wolff J. K. Warner L. M. . (2016). Regional resources buffer the impact of functional limitations on perceived autonomy in older adults with multiple illnesses. Psychol. Aging 31, 139–148. doi: 10.1037/pag0000064, 26691299

[ref28] Sinha P. Calfee C. S. Delucchi K. L. (2021). Practitioner’s guide to latent class analysis: methodological considerations and common pitfalls. Crit. Care Med. 49, e63–e79. doi: 10.1097/CCM.0000000000004710, 33165028 PMC7746621

[ref29] Skaff M. M. Pearlin L. I. (1992). Caregiving: role engulfment and the loss of self. The Gerontologist 32, 656–664. doi: 10.1093/geront/32.5.656, 1427278

[ref30] Walsh F. (2003). Family resilience: a framework for clinical practice. Fam. Process 42, 1–18. doi: 10.1111/j.1545-5300.2003.00001.x, 12698595

[ref31] Walsh F. (2016). Family resilience: a developmental systems framework. Eur. J. Dev. Psychol. 13, 313–324. doi: 10.1080/17405629.2016.1154035

[ref32] Wang Y. J. Li Z. X. Gu H. Q. Zhai Y. Jiang Y. Zhou Q. . (2022). China stroke statistics : An update on the 2019 report. Stroke and Vascular Neurology. 7, 415–450. doi: 10.1080/17405629.2016.1154035 35443985 PMC9614174

[ref33] Wang L.-J. Mu L.-L. Li X.-Y. Song P.-P. Pan Z.-Z. Gao Y.-X. . (2025). Mediating role of depression and gender in the relationship between daily video games exposure and internet gaming disorder (IGD) among college students. BMC Psychol. 13:965. doi: 10.1186/s40359-025-03328-1, 40859331 PMC12379333

[ref34] Yang X. Wang H. Chen S. He Y. Chen Y. (2025). Exploring the caregiving perceptions and needs of family caregivers of older stroke patients: a descriptive qualitative study. Int. J. Qual. Stud. Health Well-being 20:2588908. doi: 10.1080/17482631.2025.2588908, 41292099 PMC12677149

[ref35] Zhang W. Ye M.-M. Gao Y.-J. Zhou L.-S. (2023). Dyadic profiles of family resilience among patients with first-episode stroke: a longitudinal study of the first 6 months after stroke. J. Clin. Nurs. 32, 3672–3681. doi: 10.1111/jocn.16458, 35864722

[ref36] Zimet G. D. Powell S. S. Farley G. K. Werkman S. Berkoff K. A. (1990). Psychometric characteristics of the multidimensional scale of perceived social support. J. Pers. Assess. 55, 610–617. doi: 10.1080/00223891.1990.9674095, 2280326

